# Bacterial profile of diabetic foot infections and antibiotic susceptibility in a specialized diabetes centre in Cameroon

**DOI:** 10.11604/pamj.2022.42.52.31042

**Published:** 2022-05-18

**Authors:** Mesmin Dehayem Yefou, Ahmadou Musa Jingi, Martine Claude Etoa Etoga, Francine Mendane Mekobe, Batakeh Ba Agoons, Eliane Ngassam, Eugène Sobngwi, Jean-Claude Mbanya

**Affiliations:** 1 Endocrine and Diabetology Service, Yaoundé Central Hospital, Yaoundé, Cameroon,; 2 Department of Internal Medicine and Specialties, Faculty of Medicine and Biomedical Sciences, University of Yaoundé I, Yaoundé, Cameroon,; 3 Faculty of Health Sciences, University of Bamenda, Bamenda, Cameroon,; 4 Bafang District Hospital, Bafang, Cameroon

**Keywords:** Diabetic foot infections, antibiotic susceptibility

## Abstract

**Introduction:**

bacterial profile of diabetic foot infections and the antibiotic susceptibility are essential in the prescription of empirical antibiotics before the results of cultures of deep wound samples are available. The aim of this study was to determine the microbiological profile and antibiotic susceptibility of bacteria isolated from infected diabetic foot ulcers in patients attending the Yaoundé Central Hospital, Cameroon.

**Methods:**

we retrospectively analyzed the records of patients hospitalized between 2008 and 2013 for diabetic foot infections. The main outcomes were the bacteriological profile and susceptibility patterns of isolates from positive culture of deep wound sample before antibiotherapy, carried out in the national reference laboratory. Eligible clinical records of patients were retrieved from the hospitalization registry.

**Results:**

a total of 101 patient records were analyzed. The mean age of patients was 57.1 ± 9.1 years. There were more males (64.4%), mostly with type 2 diabetes (99%), with a median duration of 9 years (IQR: 4 - 14 years). Their median blood sugar on admission was 246 mg/dL. Five percent of patients died and 23% had a major amputation. Two hundred and twenty-five (225) germs were isolated, with an average of 2.25 germs per patient. Gram-negative bacteria were more frequent (75.2%). These were mainly Morganella morganii (13.8%), Klebsiella pneumonia (12%), Escherichia coli (11.6%), Proteus spp. (10.7%), and Pseudomonas aeruginosa (8.9%). Gram-positive bacteria (24.8%) were mainly Staphylococcus aureus (9.3%), Streptococcus spp. (7.6%), and Enterococcus spp. (7.1%). Gram-negative bacteria showed a high resistance to amoxicillin-clavulanic acid (78%), fluoroquinolones (55%), and gentamycin (50%). They were susceptible to imipenem (95%), amikacin (88%), and show moderate susceptibility to third generation cephalosporins (62%). Gram-positive bacteria were susceptible to vancomycine (94%), and moderately susceptible to pristinamycine (82%) and fusidic acid (67%).

**Conclusion:**

Gram-negative bacteria were more frequently associated with diabetic foot infections, and were frequently resistant to the usually prescribed antibiotics, but remain susceptible to imipenem and amikacin. Our findings should be considered when prescribing empirical anti-biotherapy for diabetic foot infections in our setting.

## Introduction

Diabetes foot infection is a frequent complication of diabetes mellitus worldwide, and it is considered a major risk factor for lower limb amputations with a lifetime risk of 25% [1-3]. The prevalence is expected to increase worldwide, parallel to the rising burden of diabetes with the greatest impact in sub-Saharan Africa [4,5]. There is local evidence of the rising trend and burden of diabetic foot infections in Cameroon against a timid rise in the number of skilled physicians [6-8]. These local studies had small sample sizes, some were performed before 2006, and did not address the problem of bacterial profile of diabetic foot infections and antibiotic susceptibility sufficiently [6] or directly [7-9]. This is key information in diabetic patient care in Cameroon, where most health facilities are not equipped with bacterial culture techniques. There is need for such local data, which need to be updated regularly so as to optimize patient care and thereby decrease the rate of foot amputations and disability due to diabetes. This retrospective study aimed at providing consistent information on the bacterial profile and antibiotic susceptibility against which future changes will be monitored. Also, evidence-based empirical antibiotic prescription will be developed.

## Methods

**Study design, setting and participants:** we report this study according to the checklist recommended by standard for reporting observational studies (STROBE) statement [10]. We carried out a cross-sectional study of patients hospitalized between 2008 and 2013 (5-year period) for diabetic foot infections in the Endocrine and Diabetology service of the Yaoundé Central Hospital of Cameroon, a low-income setting in sub-Saharan Africa. This service serves as reference centre for the education and management of endocrine and diabetes disorders in Cameroon. It also has as vocation the training of junior specialists in general internal medicine, endocrinology-diabetology, nephrology, cardiology, neurology, medical students, and paramedical staff in clinical medicine and research. It has a capacity of 20 beds, with 7 endocrinologist and diabetologist, one pediatric endocrinologist, one nephrologist and a dedicated paramedical staff. This medical team ensures a continuous patient care, with a constant review of patients and the diagnoses regularly updated based on the clinical evolution and the available laboratory data. Other parts of the unit include a well-equipped laboratory, a foot clinic, a children diabetes clinic, nutrition unit, and a research unit.

**Variables:** the main outcomes were the bacteriological profile and susceptibility patterns of isolates from positive culture of deep wound sample before anti-biotherapy, carried out in the national reference laboratory - Centre Pasteur Cameroon (CPC), using standard aerobic techniques. Other outcome measures were death and major amputation. Antibacterial susceptibility tests were performed using dilution methods. Eligible clinical records of patients were retrieved from the hospitalization registry.

**Measurements:** potential clinical records of patients were retrieved from the admission and discharge registry. Records with a positive deep wound sample culture performed at CPC were considered for analysis. We then collected information on predefined data collecting forms on the age (years), sex, duration and type of diabetes, blood glucose on admission (mg/dL), associated cardiovascular risk factors/complications (hypertension, dyslipidemia, tobacco consumption, diabetic retinopathy, diabetic nephropathy, diabetic neuropathy, peripheral arterial disease, duration of ulcers (days), antibiotic use prior to admission, duration of admission (days), and outcome (discharge, death, and major amputation). Those who left hospital against medical advice or evaded for any reason were considered as good outcome (left the unit alive).

**Bias:** included only cases with a positive aerobic deep wound culture. At the time of this study, the reference laboratory was not performing cultures for anerobic pathogens. The results reported might be overestimated the true prevalence rate of each pathogen, thus there is a risk of reporting bias. This report should be interpreted in the light of aerobic bacteria ecology.

**Sample size:** a study of the admission trends over 8 years for diabetic foot infections in the same unit showed a prevalence of 13% between the years 2000 and 2007 [8]. Assuming that the prevalence of diabetic foot ulcers increases with the prevalence rate of diabetes, for an estimated prevalence of 15% for the study period (2008 to 2013), with an 80% power to detect the significant difference and an alpha error of 5%, the sample size needed for this study was 61 cases.

**Statistical methods:** data were analyzed using the statistical package STATA version 8 (2005). We present continuous variables as means and their standard deviation, median and inter-quartile range (IQR), and categorical variables as counts and percentages (with their 95% confidence intervals). Potential risk factors for poor outcome (major amputation) were assessed using chi-squared tests. The threshold for statistical significance was set at p < 0.05. No sensitivity or missing data analysis was carried out.

**Ethical consideration:** the study was approved by the ethical committee of the Yaoundé Central Hospital. The study was carried out according to the Helsinki declaration.

## Results

A total of 2284 patients were admitted to the service during the study period. Of these, 407 were for diabetic foot ulcers. A positive pretreatment deep wound sample culture was found in 125 patients´ records. Twenty-four were excluded because the culture was not done in the National reference laboratory. Finally, 101 records of patients with 101 positive pretreatments deep wound sample cultures were considered for analysis. The mean age of patients was 57.1 ± 9.1 years. There were more males (64.4%), mostly with type 2 diabetes (99%), with a median duration of 9 years (IQR: 4 - 14 years). Their median blood sugar on admission was 246 mg/dL (IQR: 177 - 381). Five percent (5%) of patients died and 23% were amputated (Table 1). There was no significant difference between the number of germs, fever on admission, prior anti-biotherapy, hyperglycemia, peripheral arterial disease, and the risk of poor outcome.

**Table 1:** baseline characteristics of the study population and outcome (n = 101 patients)

Age, mean ± SD (years)	57.1 ± 9.1
**Type of diabetes, n (%)**	
Type 1	1 (1)
Type 2	100 (99)
**Duration of diabetes, median, [IQR] (years)**	9 [4 - 14]
**Blood glucose on admission, median, [IQR] (mg/dL)**	246 [177 - 381]
**Vascular risk factors and complications (%)**	
Hypertension	44
Dyslipidemia	71
Tobacco consumption (recent)	20
Diabetic retinopathy	61
Diabetic nephropathy	52
Diabetic neuropathy	95
Peripheral arterial disease	38
**Duration of foot ulcer, median, [IQR] (days)**	14 [10 - 30]
**Prior antibiotic use (%)**	82
**Osteomyelitis (%)**	79
**Duration of hospitalization, median, [IQR] (days)**	31.5 [21 - 43]
**Outcome (%)**	
Death	5
Major amputations	23
IQR: Interquartile range	

Two hundred and twenty-five (225) germs were isolated, with an average of 2.25 germs per patient. One germ per patient was seen in 33% of patients, 2 germs per patient in 52%, 3 germs per patient in 12%, and 4 germs per patient in 3% of patients. Gram-negative bacteria were more frequent (75.2%). These were mainly *Morganella morganii* (13.8%), *Klebsiella pneumonia* (12.0%), *Escherichia coli* (11.6%), *Proteus spp*. (10.7%), and *Pseudomonas aeruginosa* (8.9%). Gram-positive bacteria (24.8%) were mainly *Staphylococcus aureus* (9.3%), *Streptococcus spp*. (7.6%), and *Enterococcus spp*. (7.1%) (Table 2). Gram-negative bacteria showed a high resistance to amoxicillin-clavulanic acid (78%), fluoroquinolones (55%), and gentamycin (50%). They were susceptible to imipenem (95%), amikacin (88%), and were moderate susceptible to third generation cephalosporin (62%) (Table 3). Gram-positive bacteria were susceptible to vancomycine (94%), and moderately susceptible to pristinamycine (82%) and fusidic acid (67%) (Table 4).

**Table 2:** profile of bacteria isolated from patients with diabetic foot infections (n = 101 patients)

Bacteria category	Frequency (percentage)
Number of isolates	225
Gram-negative bacteria	
*Morganella morganii*	31 (13.8)
*Klebsiella pneumoniae*	27 (12.0)
*Escherichia coli*	26 (11.6)
*Proteus spp*.	24 (10.7)
*Pseudomonas aeruginosa*	20 (8.9)
*Enterobacter spp*.	13 (5.8)
*Citrobacter spp*.	8 (3.5)
*Acinobacter spp*.	6 (2.7)
*Providencia spp*.	4 (1.8)
Others	10 (4.4)
Total	169 (75.2)
Gram-positive bacteria	
*Staphylococcus aureus*.	21 (9.3)
*Streptococcus spp*.	17 (7.6)
*Enterococcus spp*.	16 (7.1)
Others	2 (0.8)
Total	56 (24.8)

**Table 3:** antimicrobial susceptibility of Gram-negative bacteria isolated from patients with diabetic foot infections (n = 101 patients)

Antibiotic	Proportion susceptible (%)				
	*Morganella morganii*	*Klebsiella pneumoniae*	*Escherichia coli*	*Proteus spp*.	*Pseudomonas aeruginosa*
Amoxicillin-clavulanic acid	0/30 (0)	5/24 (21)	4/26 (15)	17/21(81)	-
Gentamicin	16/29 (55)	7/20 (35)	5/17 (29)	18/23 (78%)	12/19 (63)
Amikacin	30/30 (100)	20/26 (77)	21/26 (81)	21/24 (88)	15/19 (79)
Imipenem	16/19 (84)	18/19 (95)	21/21 (100)	14/15 (93)	10/11 (91)
Cefotaxime	23/27 (85)	9/17 (53)	14/25 (56)	20/22 (91)	-
Ceftazidime	24/27 (89)	9/23 (39)	14/22 (63)	23/24 (95)	14/17 (82)
Ciprofloxacin	10/21 (35)	9/26 (35)	5/25 (20)	18/23 (78)	9/15 (60)
Co-trimoxazole	5/31 (16)	2/27 (7)	4/25 (19)	12/23 (52)	1/14 (71)
Ticarcillin	12/30 (40)	26/26 (100)	2/26 (8)	12/19 (63)	6/16 (38)

The proportion susceptible represents the number of bacteria susceptible by the number of bacteria tested

**Table 4:** antimicrobial susceptibility of Gram-positive bacteria isolated from patients with diabetic foot ulcer infections (n= 101 patients)

Antibiotic	Susceptibility (%)		
	*Staphylococcus aureus*	*Streptococcus spp*.	*Enterococcus spp*.
Oxacillin	10/21 (48)	-	-
Amikacin	19/21 (90)	-	-
Vancomycin	18/21 (86)	16/16 (100)	13/13 (100)
Erythromycin	12/18 (67)	3/7 (43)	9/13 (70)
Lincomycin	11/17 (65)	-	-
Rifampicin	4/5 (80)	-	-
Pristinamycin	17/20 (85)	4/6 (67)	2/2 (100)
Fusidic acid	12/18 (67)	-	-
Co-trimoxazole	12/17 (71)	6/17 (35)	1/16 (6)

The proportion susceptible represents the number of bacteria susceptible by the number of bacteria tested

## Discussion

In this retrospective study of the bacterial profile of diabetic foot infections in patients hospitalized in a tertiary diabetes centre over a 5-year period in Cameroon, we found that infection was polymicrobial with Gram-negative aerobic bacteria more frequently associated with diabetic foot infections than Gram-positive aerobic bacteria, and frequently resistant to the usually prescribed antibiotics, but remain susceptible to imipenem and amikacin.

Local data to compare the bacterial ecology of diabetic foot infections and antibiotic susceptibility are scarce in Cameroon. There is local evidence in the rising trend in the admission rate for diabetic foot ulcers [8]. This parallels the global rising prevalence of diabetes, with the greatest impact on low-income setting [4]. Background data on diabetic foot infections was reported by Kengne *et al*. [6] on a sample of 21 positive wound cultures collected between 1999 and 2002. Proteus mirabilis was the most frequent micro-organism yielded and was regularly associated with *Staphylococcus aureus*. All the micro-organisms isolated showed high susceptibility to second-generation quinolone antibiotics and were regularly susceptible to aminoglycoside antibiotics. Compared to our study, the bacterial ecology seems to have changed with *Morganella morganii, Klebsiella pneumonia* and *Escherichia coli* being the most frequently isolated germs, which exhibit a high resistance profile to the usual antibiotics but remain susceptible to amikacin and imipenem. This could be explained by the long duration of foot ulcers before admission and a high rate of prior and indiscriminate antibiotic use before admission as suggested by our study. Even though the results of our study are contrary to what has been reported in western countries with aerobic Gram-positive bacteria especially *Staphylococcus aureus* being often more frequently isolated from diabetic foot infections [11,12], many authors have reported similar results from other parts of the world. Raja NS found 52% Gram-negative aerobic bacteria in 287 isolates in Malaysia [13], Gadepalli R *et al*. 51.4% in 183 isolates in India [14], Li *et al*. 57.5% in 551 isolates in China [15], Saseedharan *et al*. 58.5% in 289 isolates in India [16]. These authors also reported poor susceptibility of aerobic Gram-negative bacteria to commonly used antibiotics, but high susceptibility to amikacin and carbapenem. This microbiological profile is particularly found in warmer countries of Africa, Asia and the Middle East.

Although the real explanation is not known, some authors have postulated foot sweating in hot climates, the use of poor footwear, a high incidence of patient self-treatment with antibiotics, frequent foot washing, and sub-optimal perineal/hand hygiene [17]. Many of our patients who had antibiotics prior to admission were by self-medication, often with drugs bought on the street. Inadequate use of antibiotics often of poor quality has contributed to the development of resistant strains. The susceptibility observed with amikacin and imipenem could be explained by the fact that these two antibiotics are expensive and are not accessible to most patients. In the light of these studies including ours, the microbial flora of diabetic foot infections in a low-income setting is polymicrobial, mainly due to gram-negative pathogens, with varying degrees of antibiotic sensitivity across the settings. This highlights the need for local and updated data on microbial flora and antibiotic sensitivity, so as to develop an evidence-based approach to antibiotic prescription while waiting for the results of culture and sensitivity, which are often delayed or not available.

Our findings should be considered when prescribing empirical anti-biotherapy for foot infections in our setting despite the limitations. We retrospectively collected data in a tertiary diabetes unit of positive aerobic cultures only. Data on anaerobic bacteria was not systematically performed by the reference laboratory. Therefore, our data estimates cannot be generalized to non-specialized diabetes units. Also, some germs isolated are not always those responsible for infections. Data on the bacterial ecology of diabetic foot infections are lacking in Cameroon as organized diabetes units are lacking. Despite these shortcomings, this study suggests a robot portrait of the situation of diabetic foot infections in this low-income setting. The clinical scenario is that of type 2 diabetic patient, diagnosed about 10 years ago, with chronically des-equilibrated diabetes, and multiple organ involvement including an infected diabetic foot ulcer with prior antibiotic use. The most likely aerobic germs are multi-resistant gram-negative bacteria susceptible to imipenem and amikacin. The death rate of patients admitted for diabetic foot infection during our study period 2008-2013 was similar to the period 2000-2007 (5% vs 6%), but the major amputation rate increased from 16% to 23% [8]. The increase in the major amputation rate could be explained by the fact that as a reference centre the service welcomes patients from all over Cameroon, some of whom arrive with advanced lesions and major amputation remain the only option.

## Conclusion

Gram-negative bacteria were more frequently associated with diabetic foot infections and were frequently resistant to the usually prescribed antibiotics, but remain sensitive to imipenem and amikacin. The rate of major amputation has increased with a resultant reduction in the death rate. Our findings should be considered when prescribing empirical anti-biotherapy for foot infections in our setting while waiting for culture and sensitivity results.

### What is known about this topic

- Infections in diabetic foot ulcers are frequent and serious complications of ulcers and can lead to limb amputation and death;
- Bacterial profile of diabetic foot infections and the antibiotic susceptibility are essential in the prescription of empirical antibiotics before the results of cultures of deep wound samples are available;
- The microbiological characteristics of diabetic foot infections have not been extensively studied in Cameroon.

### What this study adds

- This study provides bacterial profile and antibiotic susceptibility of diabetic foot infections in Cameroon from a very large number of patients;
- The data could be used as a reference in Cameroon when prescribing an empirical antibiotic for patients with diabetic foot infections.

## References

[1] Boulton AJM, Vileikyte L, Ragnarson-Tennvall G, Apelqvist J (2005). The global burden of diabetic foot disease. Lancet.

[2] Jeffcoate WJ, Harding KG (2003). Diabetic foot ulcers. Lancet.

[3] Singh N, Armstrong DG, Lipsky BA (2005). Preventing foot ulcers in patients with diabetes. JAMA.

[4] International Diabetes Federation (2019). Diabetes atlas, Brussels, Belgium, IDF.

[5] Abbas ZG, Archibald LK (2005). Epidemiology of the diabetic foot in Africa. Med Sci Monit.

[6] Kengne AP, Choukem SP, Dehayem YM, Simo NL, Feuzeu LL, Mbanya JC (2006). Diabetic foot ulcers in Cameroon: can microflora prevalence inform probabilistic antibiotic treatment?. J Wound Care.

[7] Kengne AP, Dzudie AI, Fezeu LL, Mbanya JC (2006). Impact of secondary foot complications on the inpatient department of the diabetes unit of the Yaoundé Central Hospital. Int J Low Extreme Wounds.

[8] Kengne AP, Djouogo CFT, Dehayem YM, Feuzeu L, Sobngwi E, Lekoubou A, Mbanya JC (2009). Admission trends over 8 years for Diabetic foot ulceration in a specialized Diabetes unit in Cameroon. Int J Low Extreme Wounds.

[9] Kihla AJ, Ngunde PJ, Evelyn MS, Gerard N, Ndip RN (2014). Risk factors for wound infection in health care facilities in Buea, Cameroon: aerobic bacterial pathogens and antibiogram of isolates. Pan Afr Med J.

[10] David Moher, Alessandro Liberati, Jennifer Tetzlaff, Douglas G Altman, PRISMA Group (2009). Preferred reporting items for systematic reviews and meta-analysis: the PRISMA statement. Annals of Internal medicine.

[11] Citron DM, Goldstein EJC, Merriam CV, Lipsky BA, Abramson MA (2007). Bacteriology of moderate to severe diabetic foot infections and in vitro activities of microbial agents. J Clin Microbiol.

[12] Macdonald KE, Jordan CY, Crichton E, Barnes JE, Harkin GE, Hall LML, Jones JD (2020). A retrospective analysis of diabetic foot infections at a Scottish tertiary hospital. BMC Infect Dis.

[13] Raja NS (2007). Microbiology of diabetic foot infections in a teaching hospital in Malaysia: a retrospective study of 194 cases. J Microbiol Immunol Infect.

[14] Gadepalli R, Dhawan B, Sreenivas V, Kapil A, Chaudhry R (2006). A clinico-microbiological study of diabetic foot ulcers in Indian tertiary care hospital. Diab Care.

[15] Li X, Qi X, Juan G, Ju S, Yu Z, Deng W (2018). Microbiological profile and clinical characteristics of diabetic foot infection in northern China: a retrospective multicentre survey in the Beijing area. Journal of Medical Microbiology.

[16] Saseedharan S, Sahu M, Chaddha R, Pathrose E, Bal A, Bhalekar P, Sekar P, Krishnan P (2018). Epidemiology of diabetic foot infections in a reference tertiary hospital in India. Braz J Microbiol.

[17] Uckay I, Aragon-Sanchez J, Lew D, Lipsky BA (2015). Diabetic foot infections: what have we learned in the last 30 years?. International Journal of Infectious Disease.

